# Effect of Methionine Hydroxy Analog on Hu Sheep Digestibility, Rumen Fermentation, and Rumen Microbial Community In Vitro

**DOI:** 10.3390/metabo13020169

**Published:** 2023-01-23

**Authors:** Shujie Li, Hanfang Zeng, Changjian Wang, Zhaoyu Han

**Affiliations:** College of Animal Science and Technology, Nanjing Agricultural University, Nanjing 210095, China

**Keywords:** methionine hydroxy analog, fermentation parameters, rumen microbiota

## Abstract

This experiment was conducted to evaluate the effects of a methionine hydroxy analog (MHA) on in vitro gas production, rumen fermentation parameters, and rumen microbiota. Two different MHA, 2-hydroxy-4-(methylthio) butanoic acid isopropyl ester (HMBi) and the calcium salt of the hydroxy analog of methionine (MHA-Ca), were selected for in vitro experiments. The treatments were the Control group (0% of MHA), HMBi group (2%HMBi), and MHA-Ca group (2%MHA-Ca). Dry matter digestibility was measured after 12 h and 24 h of fermentation, and fermentation parameters and microbial composition were analyzed after 24 h. HMBi and MHA-Ca showed increased (*p* = 0.001) cumulative gas production in 3 h. The total volatile fatty acids, microbial protein (MCP) concentration, acetate, and acetate to propionate ratio in the HMBi and MHA-Ca groups were significantly higher than those in the Control group (*p* = 0.006, *p* = 0.002, *p* = 0.001, *p* = 0.004), and the NH_3_-N concentrations in the HMBi and MHA-Ca groups were significantly lower than those in the Control group (*p* = 0.004). The 16S rRNA sequencing revealed that the HMBi group had a higher (*p* = 0.039, *p* = 0.001, *p* = 0.027) relative abundance of Bacteroidetes, Firmicutes, and Synergistetes and a lower relative abundance of Proteobacteria (*p* = 0.001) than the Control group. At the genus level, *Prevotella* abundance was higher (*p* = 0.001), while *Ruminobacter* abundance was lower (*p* = 0.001), in the HMBi and MHA-Ca groups than in the Control group. Spearman’s correlation analysis showed that the relative abundance of *Prevotella_1*, *Streptococcus*, and *Desulfovibrio* was positively correlated with dry matter digestibility, MCP, and fermentation parameters. MHA, thus, significantly increased gas production and altered the rumen fermentation parameters and microbiota composition of sheep.

## 1. Introduction

Methionine (Met) is the first or second limiting amino acid in ruminants, and it affects the lactation performance of dairy cows and the growth performance of sheep [1,2]. Therefore, a lack of methionine limits the performance of high-yielding ruminants. Due to the degradation of amino acids in the diet by rumen microorganisms, such as deamination by protozoa and bacteria, it is difficult to use the direction addition of Met to the diet of ruminants to regulate the level of limiting amino acids reaching the small intestine [3,4]. One strategy is to add rumen-protected methionine to protect methionine from maintaining a stable state and release it to the small intestine, thereby meeting the Met nutritional needs of the host animal.

Methionine hydroxy analogs (MHA) are the most commonly used rumen-protected methionines [5]. The 2-hydroxy-4-(methylthio) butanoic acid isopropyl ester (HMBi) and calcium salt of the hydroxy analog of methionine (MHA-Ca) are two different forms of MHA. HMBi is formed by the esterification of 2-hydroxy-4-methylthiobutyric acid with isopropanol [6]. MHA-Ca is prepared by reacting hydroxy methionine with calcium carbonate or calcium hydroxide [7]. A previous report showed that dietary supplementation with HMBi improved the growth performance and feed efficiency of beef cattle [8]. Extensive research has also shown that supplementation of methionine-deficient diets with MHA-Ca significantly improved nitrogen deposition [9]. Previous studies on MHA have focused more on the ruminal transit rate and growth performance, and there is limited information on the effects of MHA on rumen fermentation and microbiota [10,11]. Previous studies have shown that both acetate and propionate concentrations decreased with increasing HMBi [12]. However, another study showed that the supplementation of cow diets with HMBi increased the ruminal abundance of F. succinogenes and volatile fatty acid(VFA) concentrations [13]. The bacterial community population of cows that received MHA supplementation was not affected at the phylum level [14]. Therefore, the impact of MHA on rumen fermentation and the rumen bacterial community seems highly variable and remains unclear. MHAs are partially degraded in the rumen by microorganisms, and these two different forms of MHA do not completely escape rumen microbial metabolism and may be a source of Met for rumen microorganisms [10,15]. It is unknown whether MHA can be a potential modulator of rumen fermentation or alters the rumen microbiota [16].

Rumen fermentation refers to the comprehensive process of microbial activities in the rumen, including decomposition and synthesis, which are very important for the normal metabolism of ruminants [17]. A previous study showed that 70% of daily energy requirements are provided by rumen microbial fermentation products, mainly volatile fatty acids (VFAs) [18]. The rumen microbiota is a complex system that is easily influenced by diet [19]. Diets may modulate rumen fermentation by altering the rumen microbiota. In vitro techniques have been reported to adequately mimic fermentation in vivo [20,21]. Because the fermentation of microorganisms is accompanied by the production of gas, the concentration of gas produced via rumen fermentation is positively correlated with the fermentation level of the feed substrate in the rumen. In vitro techniques are widely used to explore the effects of feed additives on ruminal fermentation [22,23]. Therefore, the objective of this study was to evaluate the effects of MHA on the in vitro feed digestibility, fermentation characteristics, and rumen microbial community of Hu sheep.

## 2. Materials and Methods

### 2.1. Substrates

Fully mixed pellets were used as the fermentation substrate, and a small feed mill was used to grind the feed into powder (finely ground to pass through a 2 mm sieve). Details of the chemical composition of the substrate are presented in Table 1. Calcium was determined by potassium permanganate titration according to the Chinese national standard GB/T 6436-92; total phosphorus (TP) was measured by the molybdenum yellow method (WFJ-7200 spectrophotometer) according to the Chinese national standard GB/T 6437-92.

### 2.2. Experimental Design and In Vitro Fermentation

The experiment used a 150 mL glass bottle covered with a rubber stopper as the artificial rumen culture bottle. Rumen fluid (800 mL) was collected from four rumen-cannulated Hu sheep before morning feeding. A filter with four layers of gauze was quickly applied, and the obtained filtrate was continuously passed into CO_2_ gas. The fermentation system included 1 g of substrate, 60 mL of buffer solution, and 20 mL of rumen fluid. Rumen fluid buffer was prepared according to the method described by Menke et al. [24]. Rumen fluid buffer consisted of 400 mL distilled water, 200 mL A solution (39 g NaHCO_3_ and 2 g NH_4_ HCO, dissolved in 1 L distilled water), 0.1 mL B solution (13.2 g CaC1_2_ 2H_2_0, 10 g MnC1_2_ 4H_2_0, 1 g CoC1_2_ 6H_2_0, and 8 g FeCl 6H_2_0, dissolved in 100 mL distilled water), 200 mL C solution (5.7 g Na_2_HP0_4_, 6.2 g KH_2_P0_4_, 0.6 g MgS0_4_ 7H_2_0, dissolved in 1 L distilled water), 1 mL resazurin solution, and 40 mL reducing agent solution (95 mL distilled water, 4 mL NaOH, and 0.625 g Na_2_S 9H_2_0). The reducing agent solution was added before mixing with the rumen fluid and placed in a 39 °C water bath, and carbon dioxide was continuously introduced until the color of the mixture became pale. 

The treatments included the Control (no additive, CON), 2%HMBi (20 mg HMBi), and 2% MHA-Ca (20 mg MHA-Ca). Sixty bottles with four replicates per time (3 h, 6 h, 9 h, 12 h, and 24 h) per treatment (CON, HMBi, and MHA-Ca) were immediately sealed after introducing anaerobic N2 for 5 s. During the in vitro fermentation process, the fermentation was stopped at 3 h, 6 h, 9 h, 12 h, and 24 h and four bottles per time were removed. The samples were divided into three parts after the pH was measured with an EL20 acidity meter and gas production measured with a gas meter (CRP500, ChenRui, Technology, Shenzhen, China). One part was 5 mL of rumen fluid, and it was placed in a 5 mL centrifuge tube and stored at −20 °C for the determination of the concentration of volatile fatty acids. Another part was placed in a 50 mL centrifuge tube and stored at −20 °C for the determination of the ammonia nitrogen, bacterial protein concentration, and methionine concentration in the rumen fluid. The third part was stored in a 5 mL centrifuge tube and stored at −80 °C for the later extraction and determination of rumen microbial DNA.

### 2.3. In Vitro Dry Matter Digestibility, Amino Acid Concentration, and Fermentation Parameters

After 24 h of fermentation, fermentation substrate residues were collected using nylon bags. After this, they were dried in an oven at 60 °C for 48 h to determine the in vitro dry matter digestibility. Volatile fatty acids (VFA) were measured using gas chromatography (Agilent 6890N, Agilent Technologies, Inc., Nanjing, China). The column used was a DB-FFAP capillary column (30 m length, 0.25 mm diameter, and 0.25 μm film thickness). The temperature of the column oven was 150 °C, the equilibration time was 3 min, the temperature of the front injection port was 220 °C, and the temperature of the front detector was 210 °C. The NH_3_-N content was measured using the indophenol method with a UV–Vis spectrophotometer (PE Lambda 35, Shanghai Pudi Biotechnology Co., Ltd., Shanghai, China) at 560 nm wavelength. The microbial protein (MCP) concentration was determined using the Coomassie brilliant blue G250 staining method. Amino acid composition was analyzed using a Hitachi Automatic Amino Acid Analyzer (L-8900).

### 2.4. DNA Extraction and Sequencing

Microbial DNA was extracted using the HiPure Soil DNA Kits (or HiPure Stool DNA Kits) (Magen, Guangzhou, China) according to the manufacturer’s protocols. The full-length 16S rDNA was amplified using PCR (95 °C for 2 min, followed by 35 cycles of 95 °C for 30 s, 60 °C for 45 s, and 72 °C for 90 s, with a final extension 72 °C for 10 min) using the primers 27F: AGRGTTYGATYMTGGCTCAG; 1492R: RGYTACCTTGTTACGACTT [25]. PCR reaction was carried out in a 50 μL reaction volume with TransGen High-Fidelity PCR SuperMix (TransGen Biotech, Beijing, China), 0.2 μM forward and reverse primers, and 5 ng template DNA. Amplicons were extracted from 2% agarose gels, purified using the AxyPrep DNA Gel Extraction Kit (Axygen Biosciences, Union City, CA, USA) according to the manufacturer’s instructions, and quantified using the ABI Step OnePlus Real-Time PCR System (Life Technologies, Foster City, CA, USA). Purified amplicons were pooled in equimolar amounts and paired-end sequenced (PE250) on an Illumina platform according to the standard protocols. 

### 2.5. Bioinformatic Analysis

To obtain high-quality clean reads, raw reads were further filtered according to the following rules using FASTP (http://qiime.org/, version 0.18.0, accessed on 18 November 2022) [26]: (1) remove reads containing more than 10% of unknown nucleotides (N); (2) remove reads containing less than 50% of bases with quality (Q-value) > 20. Paired-end clean reads were merged as raw tags using FLASH [27] (version 1.2.11), with a minimum overlap of 10 bp and mismatch error rates of 2%. The abundance statistics of each taxonomy were visualized using Krona [28] (version 2.6). Chao1, ACE, Shannon, Simpson, Good’s coverage, and Pielou’s evenness indices were calculated using QIIME (http://qiime.org/, accessed on 18 November 2022) [29] (version 1.9.1). Multivariate statistical techniques, principal coordinates analysis (PCoA) of (un)weighted UniFrac, Jaccard, and Bray–Curtis distances were generated using the R project Vegan package [30] (version 2.5.3) and plotted in the R project ggplot2 package (version 2.2.1). KEGG pathway analysis of OTUs/ASV was performed using Tax4Fun [31] (version 1.0) or PICRUSt [32] (version 2.1.4). The linear discriminant analysis effect size (LEfSe) was used to identify biomarkers with significant differences in each treatment group.

### 2.6. Statistical Analysis

Data of dry matter digestibility, methionine concentration, and fermentation parameters were analyzed based on analysis of variance (ANOVA) using a general linear model in SPSS software (SPSS v. 20, SPSS Inc., Chicago, IL, USA). Rumen bacterial species among groups were compared using Tukey’s HSD test in the R project Vegan package (version 2.5.3). Alpha index comparisons among the three groups were performed using Tukey’s HSD test. We used PICRUSt to predict the KEGG functions and pathways. The analysis of variance (ANOVA) and Tukey’s HSD test were carried out to test for significant variation in the predicted KEGG pathways (%); *p* < 0.05 was defined as statistical significance.

## 3. Results

### 3.1. Changes in Fermentation Parameters

As shown in Table 2, the total volatile fatty acids (TVFAs), MCP concentrations, acetate, and acetate to propionate ratio (A:P) in the HMBi and MHA-Ca groups were significantly higher than those in the Control group (*p* = 0.006, *p* = 0.002, *p* = 0.001, *p* = 0.004), and the NH_3_-N concentrations in the HMBi and MHA-Ca groups were significantly lower than those in the Control group (*p* = 0.004). There was no significant difference in propionate, butyrate, valerate, isobutyrate, or isovalerate concentration between the three groups.

### 3.2. Effect of Methionine Hydroxy Analog on In Vitro Fermentation Cumulative Gas Production and pH

**Figure 1:**
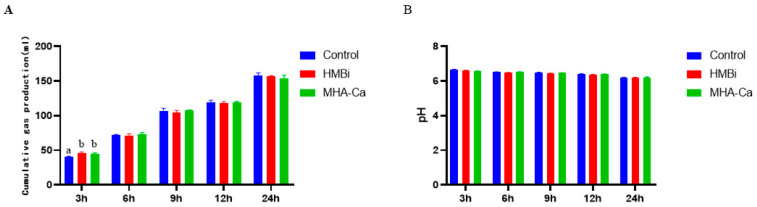
Cumulative gas production at different time (3 h, 6 h, 9 h, 12 h, and 24 h) intervals (**A**); pH at different time (3 h, 6 h, 9 h, 12 h, and 24 h) intervals (**B**). The same letter indicates no significant difference between the two groups at *p* < 0.05. Different letters between groups indicate a significant difference at *p* < 0.05.; *n* = 4 per treatment per time.

Cumulative gas production and pH were measured in each fermentation glass bottle after 3 h, 6 h, 9 h, 12 h, and 24 h of incubation (Figure 1A). Cumulative gas production was higher in the HMBi and MHA-Ca groups than in the CON groups at 3 h. No significant difference in cumulative gas production was observed at other time points. The pH did not differ (*p* > 0.05) among the three groups (Figure 1B).

### 3.3. Changes in Dry Matter Digestibility and Methionine Concentration

**Figure 2:**
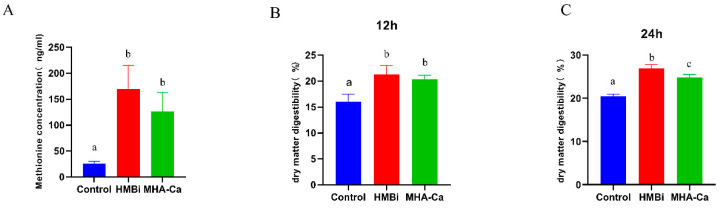
Methionine concentration at 24 h (**A**); dry matter digestibility at 12 h (**B**); dry matter digestibility at 24 h (**C**). The same letter indicates no significant difference between the two groups at *p* < 0.05. Different letters between groups indicate a significant difference at *p* < 0.05; *n* = 4 per treatment per time.

Dry matter digestibility and methionine concentration results are shown in Figure 2. Compared with that in the CON group, a significant increase in methionine concentration (*p* < 0.01) was observed in the HMBi and MHA-Ca groups (Figure 2A). Dry matter digestibility at 12 h and 24 h was higher in both HMBi and MHA-Ca groups than in the CON group (Figure 2B,C).

### 3.4. Alteration in the Composition of Rumen Bacterial Community

**Figure 3:**
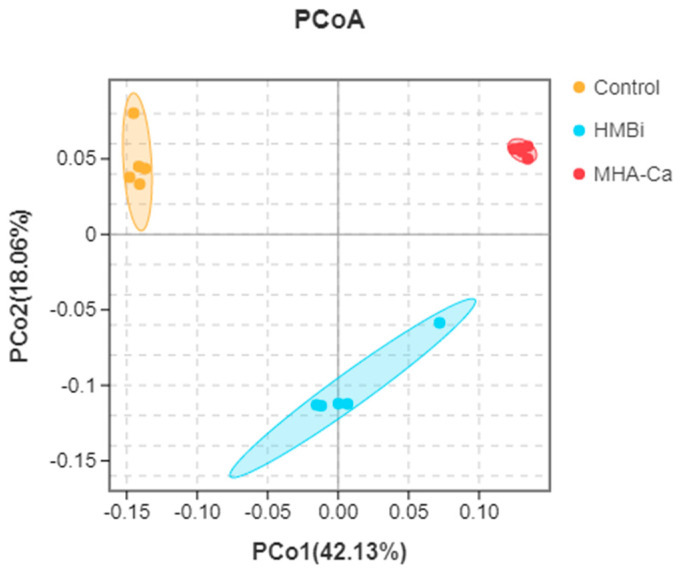
Principal coordinate analysis (PCoA) plots of the Bray-Curtis dissimilarities for the bacterial community of rumen samples. *n* = 5 per treatment per time.

A total of 1,922,646 sequences were collected from the rumen fluid samples of the three treatment groups. A total of 29,070 OTUs based on 97% sequence similarity were generated. Our results revealed that the number of observed OTUs, Chao 1 value, and abundance-based coverage estimator (ACE) were lower (*p* = 0.04, *p* = 0.04, *p* = 0.04, respectively) in the HMBi and MHA-Ca groups than in the CON group (Table 3). Compared with the Control and HMBi groups, a significant decrease was observed in the MHA-CA group. The results of the principal coordinate analysis (PCoA) profile revealed that the plots in the CON and TR groups were definitely detached (Figure 3; axis 1 + axis 2 = 60.09%).

**Figure 4:**
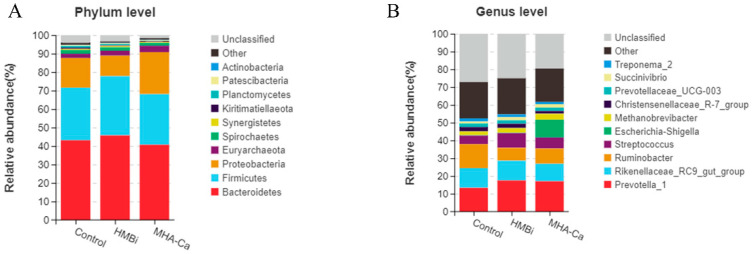
(**A**) Relative abundance of phylum species; (**B**) relative abundance of genus species.

**Figure 5:**
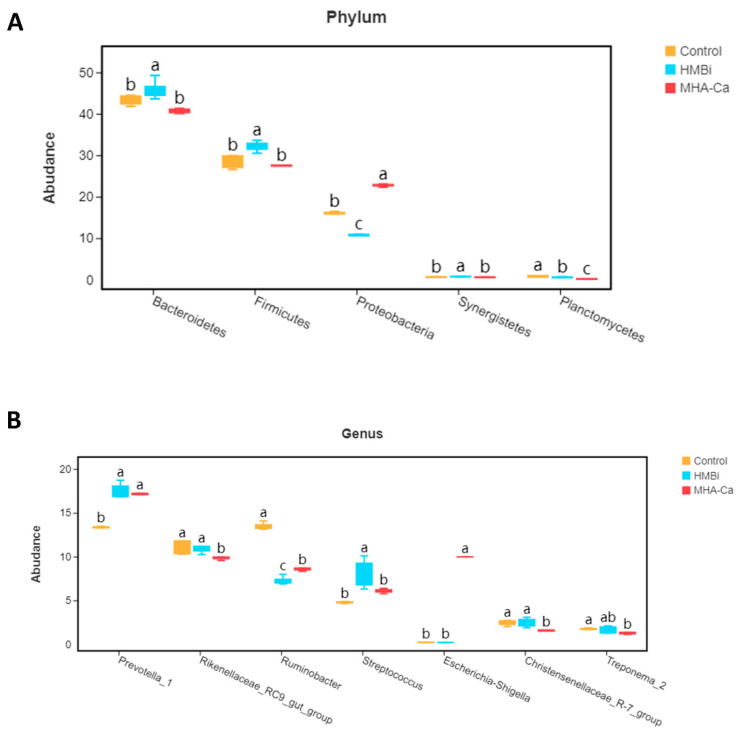
(**A**) The bacteria with significant differences among the three groups at the phylum level; (**B**) the bacteria with significant differences among the three groups at the genus level. The same letters indicate no significant difference in functional abundance between two groups; different letters indicate significant difference in functional abundance between two groups.

**Figure 6:**
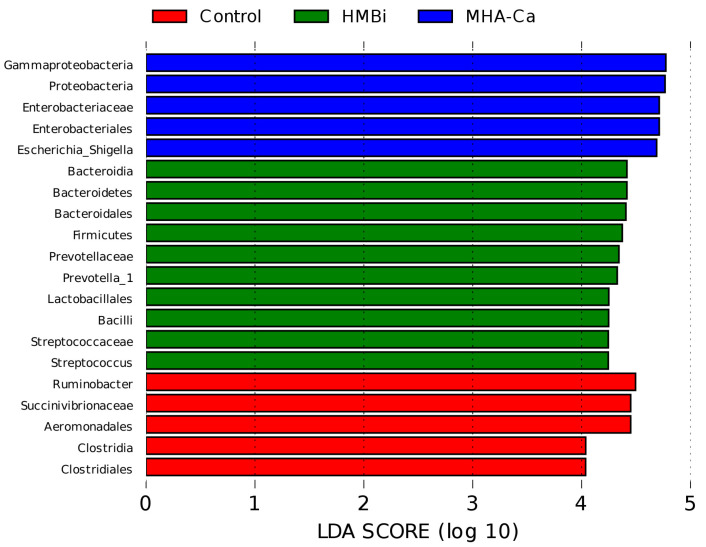
Significantly different bacterial taxa identified by the linear discriminant analysis effect size (LEfSe).

At the taxonomic level, 20 phyla, 30 classes, 46 orders, 68 families, and 151 genera were identified. The relative abundance of dominant taxa at the phylum and genus levels is shown in Figure 4A,B. At the phylum level (Figure 4A), the dominant bacteria were Bacteroidetes and Firmicutes. Bacteroidetes content was the highest in the HMBi group (49.7%) and lowest in the MHA-Ca group (46.8%). Firmicutes content was the highest in the HMBi group (32.1%) and lowest in the MHA-Ca group (27.4%). Compared to those of the Control group, the HMBi group had a higher (*p* = 0.039, *p* = 0.001, *p* = 0.027) relative abundance of Bacteroidetes, Firmicutes, and Synergistetes and a lower relative abundance of Proteobacteria (*p* = 0.001) (Figure 5A). At the genus level (Figure 4B), 71 genera had a relative abundance greater than 0.1%. *Prevotella_1*, *Rikenellaceae_RC9_gut_group*, *Ruminobacter*, and *Streptococcus* were the dominant genera in all three groups. *Prevotella* taxa were higher (*p* = 0.001), while *Ruminobacter* taxa were lower (*p* = 0.001), in the HMBi and MHA-Ca groups than in the Control group (Figure 5B). Moreover, a higher relative abundance of *Streptococcus* was observed in the HMBi group compared to the Control group. LEfSe of samples between groups revealed (Figure 6) that there were 5, 10, and 5 biomarkers that significantly differed (LDA score > 3) in the CON, HMBi, and MHA-Ca groups, respectively, similar to the results of ANOVA, with significant differences between groups.

### 3.5. Predicted Functions of Ruminal Bacterial Microbiota

**Figure 7:**
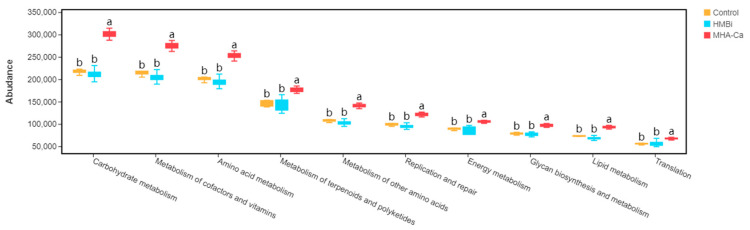
Relative abundance of the phylogenetic investigation of communities via the reconstruction of unobserved state (PICRUSt)-predicted metabolic pathways of ruminal bacterial microbiome in Control, HMBi, and MHA-Ca groups. The same letter indicates no significant difference between the two groups at *p* < 0.05. Different letters between groups indicate a significant difference at *p* < 0.05.

This study identified 29 gene families in rumen fluid samples. The gene families of carbohydrate, cofactor and vitamin, amino acid, terpenoid and polyketide, and other amino acid metabolisms were the most abundant gene families in the rumen microbiota of the three groups (Figure 7). Furthermore, the abundance of these gene families in the ruminal microbiome was significantly different among the three groups. Compared with those in the CON group, the gene families involved in carbohydrate, cofactor and vitamin, and amino acid metabolisms significantly increased (*p* = 0.001, *p* = 0.001, and *p* = 0.001, respectively) in the MHA-Ca group.

### 3.6. Interaction of Rumen Microorganisms with In Vitro Fermentation Parameters and Digestibility

**Figure 8:**
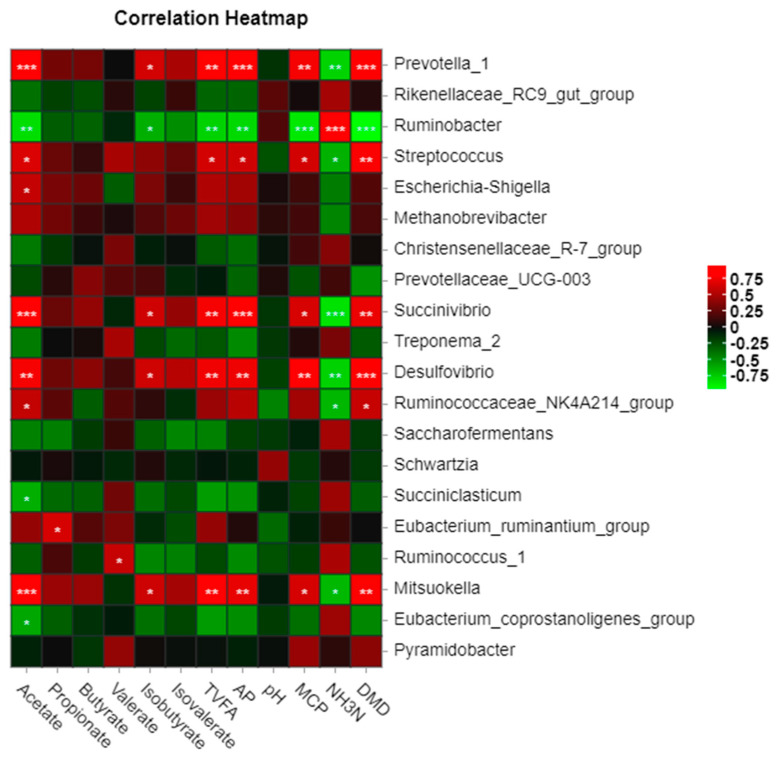
Correlation of bacterial genera with fermentation parameters and dry matter digestibility. Note: DMD, dry matter digestibility; GP, gas production; TVFA, total volatile fatty acids; AP, ratio of acetic acid to propionic acid; * *p* < 0.05, ** *p* < 0.01, *** *p* < 0.001.

A correlation heat map between the fermentation parameters, dry matter digestibility, and microbial bacteria at the genus level in vitro was constructed (Figure 8). *Prevotella_1*, *Streptococcus,* and *Desulfovibrio* were significantly positively correlated with dry matter digestibility (DMD), MCP, and fermentation parameters (*p* < 0.05). However, *Ruminobacter* showed a negative correlation with DMD and MCP (*p* < 0.05). Two bacterial genera (*Succiniclasticum* and *Eubacterium_coprostanoligenes_group*) were negatively correlated (*p* < 0.001) with TVFA and acetate.

## 4. Discussion

### 4.1. Effect of MHA on Gas Production, Dry Matter Digestibility, and Amino Acid Concentration 

MHA is a good source of Met for ruminants; approximately 50% of MHA in the diet is rapidly absorbed by the rumen wall after it reaches the rumen [33]. The remaining MHA is degraded into HMB and isopropyl ester by microorganisms in the rumen; 50-90% of the HMB is degraded in the rumen to synthesize MCP, and the other part is absorbed in the small intestine [15,16]. In this study, a significant increase in Met concentration was observed in the HMBi and MHA-Ca groups. This is consistent with previous findings, which showed that the addition of MHA increased the ruminal Met concentration [15]. Studies have also shown that rumen-administered MHA significantly increased the ruminal microbial Met concentration [34]. This result might be explained by the fact that MHA could provide a stable supply of Met for rumen bacteria to synthesize MCP [35]. Gas production is an important indicator reflecting the degree of rumen fermentation. The stronger the fermentation of the diet, the higher the microbial activity [36,37]. Previous studies have shown that the addition of rumen-protected methionine to the diet could significantly increase gas production [38,39]. Similar results were found in the present study; the cumulative gas production was higher in the HMBi and MHA-Ca groups than in the CON group at 3 h. However, at other time points, there were no significant differences in gas production among the three groups. This finding suggests that MHA promotes rumen fermentation during the early stage of fermentation. The DMD of the diet in the rumen is an important indicator of the degree of dietary nutrient utilization by the animal body and reflects the ease of digestion and utilization of the feed material [40]. Previous studies have shown that the supplementation of MHA to sheep diets improved dietary DMD [41]. In addition, studies have shown that rumen-protected methionine improved the digestibility of dietary crude protein [42]. In the present study, DMD at 12 h and 24 h was higher in both the HMBi and MHA-Ca groups than in the CON group. A possible reason for this result is that MHA provides stable Met, enhances the vitality of microorganisms, and speeds up fermentation.

### 4.2. Rumen Fermentation Characteristics

Rumen is the main location for digestion activities in ruminants, and short-chain fatty acids (SCFAs) can be produced via the fermentation of carbohydrates by microorganisms in the rumen of ruminants [43]. Short-chain fatty acids (SCFAs) are a major energy source and are important for the health and growth of ruminants [44,45]. Several reports have shown that the daily administration of 0, 4, 6, and 8 g DL-methionine into the rumen of goats using a fistula increased the average concentrations of acetate acid, propionic acid, and butyric acid, thereby increasing the average concentration of total VFA by 13.3–38.3% [46]. Studies have shown that MHA increased the ratio of acetic acid to butyric acid in the rumen contents [5,10]. Chung et al. showed that 0.52% methionine increased the content of rumen butyric acid, and 0.26% methionine had no effect on the content of VFA [47]. However, the study showed that total volatile fatty acids did not change significantly with increasing MHA supply [48]. In the present study, the effects of MHA supplementation on rumen fermentation in vitro were evaluated. The total volatile fatty acids (TVFAs), acetate, and acetate to propionate ratio (A:P) in the HMBi and MHA-Ca groups were significantly higher than those in the Control group. These results showed that the effect of MHA on rumen VFA content is unpredictable. The amount and form of methionine added may be the main factors affecting VFA content while Met is being decomposed into NH_3_-N and organic compounds by microorganisms in the rumen. Met can also be directly absorbed and utilized by microorganisms to synthesize MCP; therefore, the utilization of methionine by microorganisms also affects the production of VFA.

Rumen fluid NH_3_-N is mainly produced by the degradation of protein nitrogen and non-protein nitrogen in the chyme, and it is mainly used to synthesize MCP, which is an important indicator characterizing the activity of microorganisms [49]. Relevant studies have shown that rumen-protective methionine increased the absorption rate of ruminal NH_3_-N and reduced the concentration of ruminal NH_3_-N [50]. Previous research has shown that supplementation with RPMet could increase the MCP yield significantly in the rumen of dairy cows [51,52]. These findings were consistent with our experimental results. In this study, the NH_3_-N concentrations in the HMBi and MHA-Ca groups were significantly lower than those in the Control group. It was found that the MCP concentrations in the HMBi and MHA-Ca groups were significantly higher than those in the Control group in this research. The reason for this result may be that MHA released a small amount of methionine in the rumen to improve the rumen environment and promoted the growth of microorganisms; however, the rate at which rumen microorganisms use NH_3_-N to synthesize MCP is faster than the rate at which it decomposes dietary protein to generate NH_3_-N, resulting in a decrease in NH_3_-N concentration and an increase in MCP in the rumen.

### 4.3. Rumen Microbiota

After comparing DMD and fermentation parameters, 16S rRNA sequencing was performed. Our results revealed that the number of observed OTUs, Chao 1 value, and abundance-based coverage estimator (ACE) were lower in the HMBi and MHA-Ca groups than in the CON group. In general, the observed OTU, Chao1, and ACE indices were mainly related to the species richness of the samples [53]. This result indicates that the addition of MHA led to a decrease in the diversity of the microbial community, corroborating the results of previous studies. Previous studies have shown that deficiencies in some amino acids, such as methionine, may reduce the growth of rumen microorganisms [2], and insufficient Met supply may hinder the growth and reproduction of rumen microorganisms [54]. The reason for this conflict might be that the growth environment in the rumen could not be completely simulated due to the differences in the in vitro environment. In addition, we found that some microbial communities were altered. At the phylum level, we found that Bacteroidetes and Firmicutes were the dominant bacteria in the present study. The HMBi group had a higher relative abundance of Bacteroidetes and Firmicutes than the Control group. Previous studies showed that Bacteroidetes played an important role in the degradation and fermentation of plant structural polysaccharides in feed [55]. Moreover, Firmicutes have been reported to be the major flora involved in the fermentation of complex polysaccharides [56]. At the genus level, *Prevotella* taxa were more abundant in the HMBi and MHA-Ca groups than in the Control group. *Prevotella* has been reported to be primarily involved in the degradation of proteins and carbohydrates [57]. It was found that *Prevotella_1* was significantly positively correlated with DMD, MCP, and fermentation parameters. Based on the predicted functions of the ruminal bacterial microbiota, the gene families involved in carbohydrate, cofactor and vitamin, and amino acid metabolisms significantly increased in the MHA-Ca group compared with those in the CON group. As mentioned above, the MCP, methionine concentration, and total volatile fatty acids of the HMBi and MHA-Ca groups increased. Combined with the changes in microbial community, we could speculate that MHA was stably released into the rumen fluid and used excess N to form Met, affecting the microbial flora Bacteroidetes and Firmicutes to promote the synthesis of MCP, accelerating the digestion of carbohydrates, and promoting rumen fermentation.

## 5. Conclusions

In conclusion, this study investigated the responses of rumen fermentation parameters, rumen microbes, and dry matter digestibility to increasing MHA by an in vitro approach. The results showed that supplementation with MHA significantly improved the dry matter digestibility and promoted gas production during the early stage of fermentation. Moreover, the bacterial community composition and ruminal fermentation were altered.

## Figures and Tables

**Table 1:** Composition and nutrient levels of the substrate (DM basis, %).

Ingredients	Content (%)	Chemical Composition	Content
Corn	30.8	DM(%)	87.19
Soybean meal	5.8	ME/(MJ/kg)	16.91
Peanut meal	3.0	CP(%)	14.61
Bean straw	24.0	NDF(%)	49.71
Corn germ meal	15.0	ADF(%)	13.04
Rice husk	5.0	EE(%)	4.71
Wheat middling	2.0	Ash(%)	10.06
Molasses	1.0	Ca(%)	1.08
Malt root	6.0	P(%)	0.62
NH_4_Cl	0.4		
Limestone	2.0		
Premix	5.0		
Total	100		

The premix provided the following per kg of the diet: VA 16,000 IU, VD 25,000 IU, VE 400000IU, Fe 35 mg, Cu 20 mg, Zn 65 mg, Mn 40 mg, I 0.2 mg, Se 0.3 mg, Co 0.15 mg; DM, dry matter; CP, crude protein; NDF, neutral detergent fiber; ADF, acid detergent fiber; ME, digestible energy; EE, ether extract; metabolic energy and digestible energy were calculated, and other values were measured.

**Table 2:** Effects of MHA on rumen fermentation parameters.

Item	CON	HMBi	MHA-Ca	SEM	*p*-Value
NH3N (mg/dL)	10.18 ^a^	7.99 ^b^	7.96 ^b^	0.37	0.004
MCP (mg/mL)	0.46 ^b^	0.59 ^a^	0.55 ^a^	0.01	0.002
Total VFA (mmol/L)	37.61 ^b^	44.69 ^a^	45.99 ^a^	1.34	0.006
Acetate	21.56 ^b^	26.61 ^a^	27.86 ^a^	0.91	0.001
Propionate	9.65	10.01	10.36	0.22	0.482
Butyrate	1.31	1.53	1.66	0.12	0.528
Valerate	2.99	3.23	2.80	0.14	0.530
Isobutyrate	1.50	2.54	2.57	0.23	0.083
Isovalerate	0.58	0.77	0.73	0.05	0.265
Acetate/propionate (A:P)	2.24 ^b^	2.66 ^a^	2.69 ^a^	0.07	0.004

The same letter indicates no significant difference between the two groups at *p* < 0.05. Different letters between groups indicate a significant difference at *p* < 0.05; *n* = 4 per treatment per time.

**Table 3:** α-Diversity of microbial communities based on OTU level.

Index Type	Control	HMBi	MHA-Ca	SEM	*p*-Valve
Observed OTU	2173.6 ^a^	1887.4 ^b^	1389.8 ^c^	95.4	0.04
Shannon	7.53 ^a^	7.31 ^a^	6.65 ^b^	0.11	0.06
Simpson	0.97	0.97	0.96	0.01	0.06
Chao1	2232.4 ^a^	1950.6 ^b^	1458.8 ^c^	93.9	0.04
ACE	2309.5 ^a^	2022.9 ^b^	1517.6 ^c^	95.9	0.04

The same letter indicates no significant difference between the two groups at *p* < 0.05. Different letters between groups indicate a significant difference at *p* < 0.05. ^a–c^ Means having different superscripts in the same row differ significantly (*p* < 0.05).

## Data Availability

The raw reads were deposited in the NCBI Sequence Read Archive (SRA) database (Accession Number: PRJNA898761).
